# Regulating the Regulator: Phosphorylation of PKC θ in T Cells

**DOI:** 10.3389/fimmu.2012.00227

**Published:** 2012-08-01

**Authors:** Michael Freeley, Aideen Long

**Affiliations:** ^1^Clinical Medicine, Trinity College DublinDublin, Ireland

Protein kinase C θ (PKC θ) is a serine/threonine kinase that is now firmly established as a central component in T cell activation, proliferation, differentiation, and apoptosis (Hayashi and Altman, 2007). Since it was first discovered that PKC θ re-localizes to the immunological synapse (IS) in conventional effector T cells following T cell stimulation, many roles have now been defined for this kinase in these cells such as (a) activation of NF-κB, AP-1, and NFAT transcription factors that control the synthesis of pro-inflammatory cytokines and the anti-apoptotic molecule Bcl-x_L_ (Hayashi and Altman, 2007), (b) regulation of IS dynamics (Sims et al., 2007), (c) up-regulation and clustering of the integrin LFA-1 on the T cell surface (Tan et al., 2006; Letschka et al., 2008) – thus facilitating stable adhesion between T cells and antigen-presenting cells (APC) and/or migration into inflamed tissues, (d) re-orientation of the microtubule-organizing center toward the APC (Quann et al., 2011), and (e) fine tuning of T cell activation by regulating the intracellular localization, degradation, and internalization of key signaling molecules (Nika et al., 2006; von Essen et al., 2006; Gruber et al., 2009). A new function for PKC θ has also recently been revealed with the finding that this kinase regulates an inducible gene expression program in T cells by associating with chromatin in the nucleus (Sutcliffe et al., 2011).

A host of studies have now convincingly demonstrated that targeting PKC θ could be a viable therapeutic strategy to block the T cell inflammatory response in autoimmunity, allergy, and allograft rejection (Marsland and Kopf, 2008; Zanin-Zhorov et al., 2011; Altman and Kong, 2012). For example, PKC θ-deficient mice (PKC θ^−/−^) have reduced incidence and severity of Th2 and Th17-mediated inflammatory disorders, including asthma, inflammatory bowel disease, multiple sclerosis, arthritis, and allograft rejection in comparison to their wild-type littermates (PKC θ^+/+^; Marsland and Kopf, 2008; Zanin-Zhorov et al., 2011; Altman and Kong, 2012). Intriguingly, PKCθ^−/−^ mice are still capable of mounting relatively normal Th1 and CD8^+^ T cell-mediated immune responses to infectious viruses (Marsland and Kopf, 2008; Zanin-Zhorov et al., 2011; Altman and Kong, 2012). Secondly, the recent finding that inhibition of PKC θ increases the suppressive activity of regulatory T cells (Zanin-Zhorov et al., 2010) suggests that therapeutic strategies designed to inhibit this kinase may hold great promise in diverting the pro/anti-inflammatory balance toward a reduction in inflammation in T cell autoimmunity and allergy, whilst at the same time maintaining immunity to viral pathogens. Lastly, that PKC θ has a restricted tissue expression profile and is highly expressed in T cells suggests that targeting this molecule with specific inhibitors should have minimal effects in other cells and tissues (Hayashi and Altman, 2007; Altman and Kong, 2012). In spite of all this promising data however, a number of studies have demonstrated that targeting PKC θ could potentially have some undesired effects. For example, it has been reported that CD8^+^ T cells from PKC θ^−/−^ mice have a survival defect following activation (Barouch-Bentov et al., 2005; Saibil et al., 2007; Kingeter and Schaefer, 2008). In addition, it has been reported that PKC θ^−/−^ mice have an impaired anti-leukemic response (Garaude et al., 2008), which likely results from reduced tumor surveillance *in vivo*. It is important therefore that these issues are addressed in respect of any PKC θ-targeting strategies that are developed in the future.

Although much has been learned about PKC θ in T cells, considerable gaps still exist in our knowledge as to how this kinase is regulated, including the upstream signals and interacting partners that control its intracellular localization and catalytic activation at various locations in the cell. Furthermore, although a plethora of substrates that are phosphorylated by PKC θ *in vitro* have now been characterized (Nika et al., 2006; Hayashi and Altman, 2007; Letschka et al., 2008), whether any of these are *bona fide* substrates *in vivo* remains to be addressed. Like many other kinases, PKC θ is also regulated by phosphorylation on a host of serine, threonine, and tyrosine residues that influence its activity and intracellular localization. Six phosphorylation sites have been mapped on PKC θ in T cells to date. Some of these sites appear to be phosphorylated by unrelated upstream kinases, while other sites are regulated via auto-phosphorylation. Three of these phosphorylation sites are highly conserved on most other PKC isoforms, which suggests that they may regulate aspects that are central to all isoforms, such as stability. In contrast, PKC θ contains three phosphorylation sites that appear to be unique to this isoform.^1^ Therefore PKC θ may execute distinct functions and/or be regulated differently in T cells (Freeley et al., 2011). In this issue of *Frontiers in T Cell Biology*, Wang et al. (2012) summarize the regulation of PKC θ by phosphorylation during T cell signaling. Understanding the pathways that regulate PKC θ in T cells may provide additional therapeutic targets for the treatment of inflammatory diseases.

## Footnotes

^1^Some of these three residues on PKC θ may also be found in other PKC isoforms, but their phosphorylation on other PKCs has not been described.
